# COVID-19 infection and neurodegeneration: Computational evidence for interactions between the SARS-CoV-2 spike protein and monoamine oxidase enzymes

**DOI:** 10.1016/j.csbj.2022.02.020

**Published:** 2022-02-24

**Authors:** Lucija Hok, Hrvoje Rimac, Janez Mavri, Robert Vianello

**Affiliations:** a Laboratory for the Computational Design and Synthesis of Functional Materials, Division of Organic Chemistry and Biochemistry, Ruđer Bošković Institute, Zagreb, Croatia; b Department of Medicinal Chemistry, University of Zagreb Faculty of Pharmacy and Biochemistry, Zagreb, Croatia; c National Institute of Chemistry, Ljubljana, Slovenia

**Keywords:** Brain amines, Molecular dynamics simulations, Neurodegeneration, Neurotransmitters, SARS-CoV-2 spike protein, South African B.1.351 variant

## Abstract

Although COVID-19 has been primarily associated with pneumonia, recent data show that its causative agent, the SARS-CoV-2 coronavirus, can infect many vital organs beyond the lungs, including the heart, kidneys and the brain. The literature agrees that COVID-19 is likely to have long-term mental health effects on infected individuals, which signifies a need to understand the role of the virus in the pathophysiology of brain disorders that is currently unknown and widely debated. Our docking and molecular dynamics simulations show that the affinity of the spike protein from the wild type (WT) and the South African B.1.351 (SA) variant towards MAO enzymes is comparable to that for its ACE2 receptor. This allows for the WT/SA⋅⋅⋅MAO complex formation, which changes MAO affinities for their neurotransmitter substrates, thereby impacting their metabolic conversion and misbalancing their levels. Knowing that this fine regulation is strongly linked with the etiology of various brain pathologies, these results are the first to highlight the possibility that the interference with the brain MAO catalytic activity is responsible for the increased neurodegenerative illnesses following a COVID-19 infection, thus placing a neurobiological link between these two conditions in the spotlight. Since the obtained insight suggests that a more contagious SA variant causes even larger disturbances, and with new and more problematic strains likely emerging in the near future, we firmly advise that the presented prospect of the SARS-CoV-2 induced neurological complications should not be ignored, but rather requires further clinical investigations to achieve an early diagnosis and timely therapeutic interventions.

## Highlights

• WT and the South African SARS‐CoV‐2 variant show comparable ACE2 and MAO affinities.
• Identified MAO/spike protein complexes modify MAO affinity for its neurotransmitters.
• Such changes impact metabolic clearance of brain amines and misbalance their level.
• This links MAO interference with neurological illnesses following COVID‐19 infection.
• More contagious SA variant gives larger MAO disturbances, which should not be ignored.

## Graphical abstract

**Figure:**
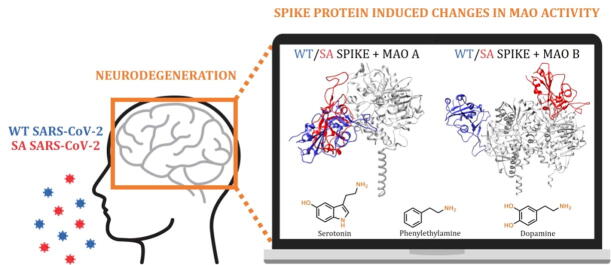

## 1 Introduction

In December 2019, a novel SARS-CoV-2 coronavirus emerged from China and spread worldwide as a pandemic, causing a public health emergency and killing over 2 million people in the first year [1], while totalling over 5.4 million fatal outcomes by the end of 2021 [2]. This infection is responsible for heterogeneous clinical disturbances, leading to severe pneumonia and the acute respiratory distress syndrome, termed COVID-19, which manifests not only as a respiratory illness but also impacts the cardiovascular, renal, and the nervous system functions [3]. Until now, this outbreak has been accompanied by a high burden on a lot of social, economic and political distress throughout the world [4] due to governmental containment measures, such as quarantine, social distancing, and lockdown. Importantly, the long-term consequences of the virus, including its effects on mental and physical health, however, might even pose a much more serious threat in the years to come.

Despite the fact that coronaviruses have not yet been linked with particular long-term neurological sequels, the occurrence of these manifestations in COVID-19 patients is becoming increasingly reported [5], [6], [7]. Although this suggests a possibly acute or a subacute neuropathogenicity of the virus, the risk of neurological complications in patients affected by the SARS-CoV-2 is still not entirely clarified [6], [7], [8], [9], and should not be ignored.

The SARS-CoV-2 is a novel virus and its pathophysiological mechanisms in various physiological systems are yet to be fully understood. However, a lot can be learnt from the other coronavirus subtypes known to infect humans [8]. A great structural similarity between the SARS-CoV-2 and beta coronaviruses suggests a hypothesis that the SARS-CoV-2 also possesses similar neurotrophic and neuroinvasive properties. Additionally, the SARS-CoV and the SARS-CoV-2 share around 80% genome similarity [10] and use the same ACE2 host receptor to infiltrate human cells [11], [12]. Apart from this role of the ACE2 receptor, gene expression studies have revealed that the *ACE2* gene shows the most significant co-expression and co-regulation with the aromatic *L*-amino acid decarboxylase, which is responsible for biosynthesis of biogenic amines and the conversion of *L*-DOPA into dopamine. This indicates that ACE2 downregulation, induced by the SARS-CoV-2 infection, might be associated with concomitant alterations in the brain amine levels, which is strongly implicated in the etiology of Alzheimer and Parkinson diseases [13]. In addition, the CT/MRI scan of COVID-19 patients showed an acute necrotizing encephalopathy, a rare encephalopathy typically associated with a viral infection of the brain tissue [14], indicating a direct CNS infection by the SARS-CoV-2. In fact, at least four known coronaviruses (HCoV-229E, HCoV-OC43, SARS-CoV, and MERS-CoV) can penetrate into the central nervous system [15], and the literature agrees that the CNS infection by the SARS-CoV-2 virus may promote a development of neurodegenerative diseases [16], [17], [18], especially in people already at risk [19]. Still, a significant difference between the SARS-CoV-2 and other coronaviruses is the longer length of the spike protein sequence [20]. This disparity has been suggested to confer a higher transmissibility potential to the SARS-CoV-2, making it possible for the virus to infect humans of different races and geographical origins [20], [21], [22]. It is proposed that the virus enters the CNS through different routes, including the olfactory and trigeminal nerves, the cerebrospinal fluid, the vasculature, and the lymphatic system [23], even without an initial lung involvement. Once the virus enters the nervous system, it can bind to the highly expressed ACE2 receptor in glial cells and neurons, and from there disseminate throughout the brain.

Clinical studies show that approximately 36% of all COVID-19 patients exhibit neurological symptoms such as stroke, headache, impaired consciousness, and paresthesia [24], but also neurobehavioral indications such as euphoria, anxiety, and depression, as well as cognitive dysfunction, especially in elderly patients, which are the most susceptible to the infection [25]. Accumulated evidence confirms the SARS-CoV-2 potential to invade the CNS, however, its effects at the molecular and mechanistic levels have so far only been speculations and hypotheses. Although a COVID-19 infection certainly represents a stressful event, which, on its own, may have a role in triggering neurodegeneration [26], in this work we used a range of computational approaches to demonstrate that the SARS-CoV-2 can initiate misbalances in the monoaminergic system by binding the monoamine oxidase enzymes, MAO A and MAO B, with affinities comparable to those for its ACE2 receptor, thus causing a significant dysregulation in the way MAOs interact with their physiological substrates. Since MAO enzymes are involved in the metabolic clearance and regulation of brain amine levels [27], [28], including neurotransmitters dopamine and serotonin, whose even the slightest disparity is strongly linked to the etiology and course of various neurological illnesses [29], [30], [31], such downregulation and modified MAO activity likely represent incipient stages of neurological disturbances, which are already broadly speculated in the literature [32], [33], [34], [35]. Importantly, a potential relationship between the MAO enzymes and the SARS-CoV-2 infection has recently been proposed by Cuperlovic-Culf, Green and co-workers [36], who used metabolomic profiling to detect a decrease in the concentration of phenylethylamine (**PEA**) metabolites within the cerebrospinal fluid and blood of COVID-19 related patients relative to healthy individuals, a trend similarly observed with more than 200 other metabolites, involving amino acids and their derivatives [37]. Knowing that MAO B preferentially degrades **PEA** in the CNS [27], the authors ascribed this observation to a possible interference of the spike protein with the substrate entrance to the MAO B active site, thus providing a justification to our hypothesis. Additionally, it allows us to be confident that our work aids in identifying the critical role of the MAO enzymes towards an increased incidence of neurological disorders in the SARS-CoV-2 infected individuals, therefore placing a neurobiological link between these two conditions in the spotlight.

## 2 Computational methods

All technical details about performed computational simulations are presented in the Supporting Information document.

## 3 Results and discussion

### 3.1 Interactions between the ACE2 receptor and the spike protein from the WT SARS-CoV-2 and its South African variant B.1.351

The SARS-CoV-2 infiltrates human cells through an interaction between the virus S1 spike protein and the ACE2 receptor, a mechanism that has been extensively studied and characterized using various structural [38], [39] and computational [40], [41], [42], [43], [44] techniques. Therefore, we felt it was useful to employ our computational setup to find relevant binding poses and dynamical features of the spike protein-ACE2 complexes and benchmark the obtained results with relevant literature data. By doing so, we have considered the wild-type (WT) virus and its B.1.351 South African (SA) variant, which is known to possess a higher ACE2 binding affinity [45], an increased transmissibility and infectivity, and more severe clinical outcomes [45], [46], [47], all of which make it a good model to discuss relative differences among strains. Therefore, after a docking analysis had suggested relevant binding poses as starting points for the molecular dynamics simulations, the latter identified the representative structure of the WT⋅⋅⋅ACE2 complex (Fig. S1) that closely matches its crystal structure [38], [39]. Importantly, the subsequent MM-GBSA analysis revealed a binding free energy among proteins of Δ*G*_BIND_ = –46.6 kcal mol^−1^ (Table 1), being in excellent agreement with –46.4 kcal mol^−1^ independently reported by Yarovski [48], Murugan [49] and their co-workers, which will serve as a reference. Also, a decomposition of the binding affinity on a *per-residue* basis underlined crucial residues in both proteins that are contributing the most to the binding (Table S1). Interestingly, the top 15 spike protein residues are responsible for around 78% of the total binding energy, and all belong to the receptor-binding motif (RBM) of the receptor-binding domain (RBD), in line with other reports [38], [39], [50], which confirms the validity of our calculations. The only exception is Lys417 with a notable contribution of –0.95 kcal mol^−1^, which forms a salt bridge with Asp30 from ACE2, as demonstrated earlier [38], [39], [48]. Also, within residues disfavouring the binding, one notices that the first two residues, Asp405 (0.99 kcal mol^−1^) and Glu406 (0.80 kcal mol^−1^), do not belong to the spike protein RBM area, while the third one, Glu484 (0.66 kcal mol^−1^), is one of the residues that is mutated in the SA variant to Lys484, where it exhibits a reduced unfavourable contribution by 0.13 kcal mol^−1^, from 0.66 kcal mol^−1^ in WT to 0.53 kcal mol^−1^ in SA (Table S1). Also, a highly unfavourable contribution from Ser19 in ACE2 (+2.42 kcal mol^−1^) agrees with the reported virus ability to improve binding upon changing its nearby environment [51].

**Table 1:** Binding free energies (Δ*G*_BIND_) among proteins studied in this work, calculated from molecular dynamics trajectories using the MM‐GBSA approach (in kcal mol^−1^).^a^

	ACE2 receptor	MAO A enzyme	MAO B enzyme
Wild-type (WT) spike protein	–46.6	–38.3	–38.1
SA B.1.351 variant spike protein	–54.8	–49.0	–62.7

a Decomposition of the obtained Δ*G*_BIND_ values on a *per-residue* basis is given in the Supporting Information.

To put these values in context, let us discuss data for a more contagious B.1.351 SA variant, first identified in South Africa in October 2020 [52]. It carries the N501Y, E484K and K417N mutations in the RBD area [53] that confer an increased antibody resistance [54]. The overlap between binding poses (Fig. S2) does not reveal any significant difference in the way both variants approach ACE2, yet the identification of specific residues governing the interaction shows insightful aspects. Similar to WT, all of the top contributing residues in SA belong, without exceptions, to the RBM area. Still, to our surprise, the most dominant residue is Tyr501, which is mutated from Asn501 in WT. Its individual contribution of –9.11 kcal mol^−1^ surpasses all WT residues and is solely responsible for around 17% of the total binding energy. Specifically, through the N501Y mutation, the SARS-CoV-2 increases the individual contribution of this residue by as much as –6.7 kcal mol^−1^, which is both highly significant and highly disturbing, knowing that this mutation is well conserved and present in the UK and Brazilian strains as well [53], although most likely independently evolved [55]. The reasons for the increased Tyr501 contribution are threefold: it forms (i) O–H⋅⋅⋅O hydrogen bonds with Asp38, (ii) cation⋅⋅⋅π interactions with Lys353, and (iii) T-shaped C–H⋅⋅⋅π contacts with Tyr41, which is, amazingly, exactly the same binding environment demonstrated in the UK variant [56], where this is the only RBM mutation. Overall, this results in a significantly higher SA affinity for ACE2, Δ*G*_BIND_ = –54.8 kcal mol^−1^ (Table 1), in line with the value of –53.7 kcal mol^−1^ reported by Magistrato and co-workers [57], which directly translates to its higher infectiveness; strongly coupled features well-demonstrated across species [42]. At this point, it is worth to stress that Δ*G*_BIND_ values obtained by this approach are somewhat overestimated in absolute terms. This is a known limitation of the MM-GBSA approach, as extensively discussed in a recent review by Homeyer and Gohlke [58], which also underlined its huge potential in predicting relative binding energies in biomolecular complexes [43], [48], [49], [58], precisely how this approach was used here. In this context, our analysis successfully reproduced the higher affinity of the SA strain, being in excellent agreement with experiments [45]. It is also interesting to observe that, despite the three mutations in the RBD area, the order of contributing residues is mostly unchanged among strains, which underlines the significance of single point mutations within this structural element, and raises concerns that further mutations might likely offer even more problematic SARS-CoV-2 variants. Overall, we can summarize that, through the three mutations (N501Y, E484K, K417N), the SA variant increases its ACE2 affinity by –5.8 kcal mol^−1^, being solely responsible for almost 70% of the overall affinity gain (–8.2 kcal mol^−1^) between the SA strain and ACE2. This strongly confirms the hypothesis that positively selected virus mutations convey benefits regarding immune evasion and viral fitness, but also for the ACE2 binding, thus contributing to the evolution rate and expectedly causing higher disturbances in the infected organisms.

### 3.2 Interactions between MAO enzymes and the spike protein from the WT SARS-CoV-2 virus and its South African variant B.1.351

**Fig. 1:**
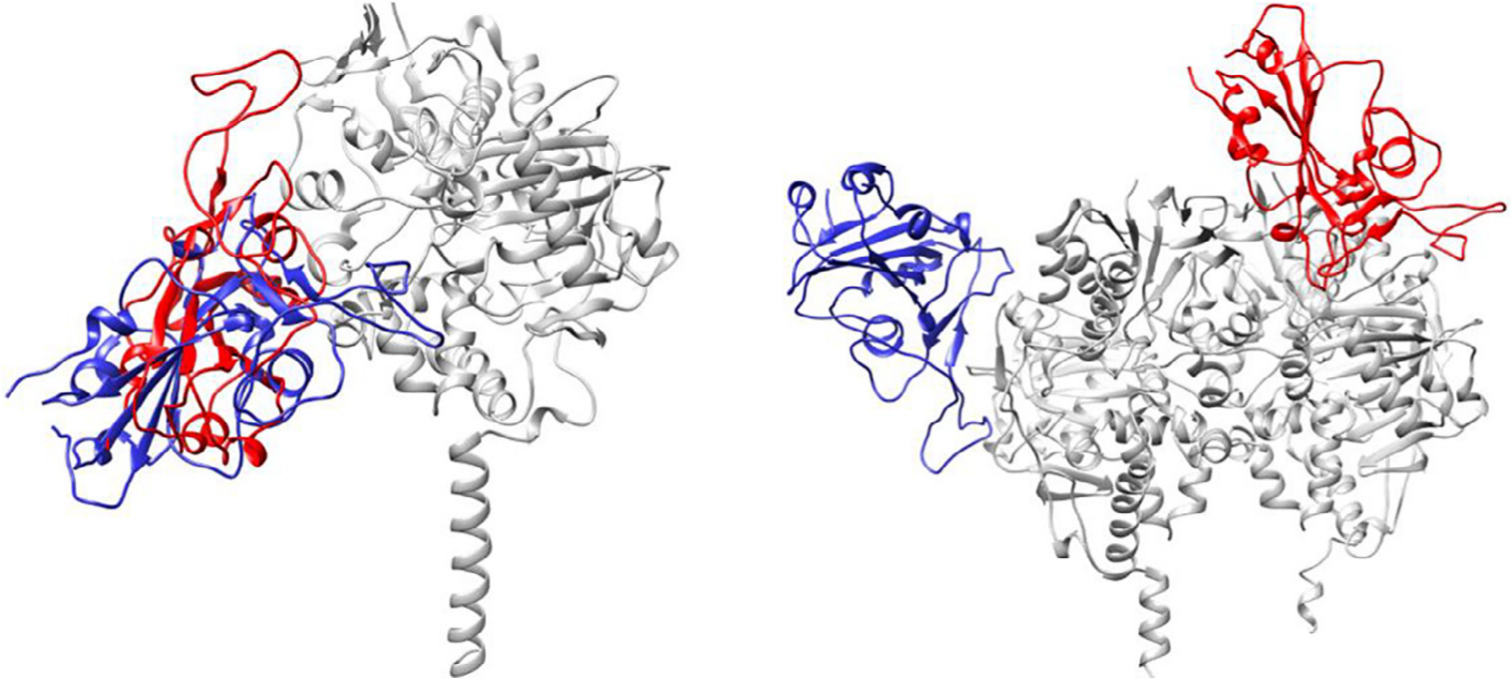
Overlap of the most favourable binding positions of the WT (in blue) and the SA (in red) spike protein in complex with the MAO enzymes (in gray), MAO A (left) and MAO B (right), as elucidated from molecular dynamics simulations.

After establishing that the WT and SA strains recognize ACE2 in almost identical ways, mainly through their RBM units, and that the SA⋅⋅⋅ACE2 complex reveals a stronger interaction, with both aspects firmly in line with experiments, we proceeded by analyzing interactions among strains and MAO isoforms. In each case, docking analysis elucidated ten most favourable binding poses (Fig. S3), which were submitted to MD simulations (for details, see Computational Details), and trajectories with the highest protein–protein affinities were used for further analysis. Elucidated representative structures are shown in Figs. S4 and S5, while the calculated affinities are given in Table 1, together with their decomposition on a *per-residue* basis in Tables S2–S3. In addition, the overlap of the resulting spike protein binding poses to each MAO isoform is depicted in Fig. 1.

As with ACE2, both SARS-CoV-2 variants predominantly interact with MAO enzymes through their RBM region. This is seen in the fact that a majority of crucial interacting residues belongs to this structural element of the spike protein. This holds especially for the WT strain, while in the SA strain, the mutated Asn417 becomes very significant in binding MAO A with a second largest individual contribution of –4.08 kcal mol^−1^. Interestingly, the WT binds both MAOs with an almost identical exergonicity, –38.3 kcal mol^−1^ for MAO A and –38.1 kcal mol^−1^ for MAO B (Table 1). Given that the stability of the WT⋅⋅⋅ACE2 complex was estimated at Δ*G*_BIND_ = –46.6 kcal mol^−1^, a difference in a few kcals mol^−1^ allows a formation of the matching WT⋅⋅⋅MAO complexes. In MAO A, the interaction with the WT spike protein (S) is dominated by S-Phe486, which establishes cation⋅⋅⋅π interactions with Lys357 (Fig. S4). This is followed by S-Ser477, which joins S-Thr478 in forming hydrogen bonds with Glu329, and by S-Thr500 that interacts with the side chain carbonyl from Gln293. It is also worth mentioning that S-Lys417 forms a salt bridge with Glu159, which imitates an analogous interaction with Asp30 from the ACE2 receptor. Considering MAO B, the relative importance in spike protein residues is reversed relative to MAO A, making S-Tyr449 the most dominant residue, which is engaged in a hydrogen bonding with Gln49, and in a T-shaped C–H⋅⋅⋅π interaction with Tyr53 (Fig. S4). Interestingly, S-Glu484, which is mutated to S-Lys484 in the SA strain, is the first in disfavouring the binding between the proteins (+1.73 kcal mol^−1^). It is placed in close vicinity of the crucial MAO B residue, cationic Arg307, yet not interacting with it, thus its unfavourable contribution.

When a more contagious SA variant is concerned, its affinity for ACE2 is higher relative to the WT, but it is remarkable that its tendency to bind both MAOs is increased as well. This is particularly interesting for MAO A, where the binding pose for the SA strain is almost identical to the one established by the WT (Fig. 1), yet the affinity is increased by 10.7 kcal mol^−1^ to Δ*G*_BIND_ = –49.0 kcal mol^−1^ (Table 1). Recalling that its affinity for ACE2 is –54.8 kcal mol^−1^, again only a few kcals mol^−1^ higher, opens a possibility that the matching SA⋅⋅⋅MAO A complex could be formed. There, the two crucial residues with individual contributions exceeding 4 kcal mol^−1^ are S-Leu455 and the mutated S-Asn417, which use their backbone carbonyl and side chain amide, respectively, to interact with Arg297 in MAO A (Fig. S5). It is worth emphasizing that all three mutations present in the SA strain are promoting MAO A binding. As mentioned, the mutated Asn417 is the second most dominant residue (–4.08 kcal mol^−1^), while its non-mutated analogue Lys417 in the WT has a significantly lower contribution (–0.39 kcal mol^−1^). Analogously, in the SA strain, Glu484 (+0.07 kcal mol^−1^) and Asn501 (+0.51 kcal mol^−1^) are replaced by Lys484 (+0.22 kcal mol^−1^) and Tyr501 (+0.07 kcal mol^−1^), respectively, indicating that all three SA mutations not only enhance the ACE2 binding, but also jointly promote the MAO A complex formation by –3.98 kcal mol^−1^, which is significant.

The situation with MAO B is even more remarkable. The interaction energy in the SA⋅⋅⋅MAO B complex is Δ*G*_BIND_ = –62.7 kcal mol^−1^ (Table 1), being the highest of all, even surpassing the stability of the matching SA⋅⋅⋅ACE2 complex by –7.9 kcal mol^−1^. This suggests that the SA variant would, following the initial ACE2 binding and cell infiltration, mainly attach to MAO B, a process that is thermodynamically very favourable, and which might appear particularly troublesome for neurological conditions. This recognition is dominated by S-Arg346, which forms hydrogen bonds with the Glu232 side chain and the Ala35 backbone carbonyl, both from the subunit B of the MAO B enzyme (Fig. S5). Such a positive pairing leads to a very high individual contribution from S-Arg346 (–6.54 kcal mol^−1^), solely contributing to around 22% of the total binding energy. One of the reasons for a high SA⋅⋅⋅MAO B binding affinity relative to the WT lies in different MAO B areas preferred by both strains (Fig. 1). While the WT position is almost exclusively located in one subunit, the SA strain is most favourably located closer to the interface between the two MAO B subunits, which allows both of them to participate in the binding, and which might be, at least partly, responsible for the increased affinity. Although our analysis identified that a majority of crucial residues belongs to the subunit B (Table S3), the most significant residue in MAO B is Arg242 belonging to the subunit A. Its very high individual contribution (–7.35 kcal mol^−1^) comes as a result of a stable salt-bridge with S-Glu340, which was persistent during MD simulations (Fig. S6). Interestingly, despite such favourable binding to MAO B, noneneither of the three mutated residues emerges among those dominant for an increased complex stability. Still, all three residues make notable contributions, as the introduced Asn417 (+0.06 kcal mol^−1^), Lys484 (–0.40 kcal mol^−1^) and Tyr501 (+0.08 kcal mol^−1^) surpass the initial WT residues Lys417 (+0.11 kcal mol^−1^), Glu484 (+1.73 kcal mol^−1^) and Asn501 (+0.16 kcal mol^−1^), thus enhancing the binding affinity by –2.26 kcal mol^−1^.

In concluding this section, let us emphasize that the affinity of both SARS-CoV-2 variants towards the MAO isoforms is very much comparable to that for their ACE2 receptor, thus indicating a feasibility and likelihood of the WT/SA⋅⋅⋅MAO A/B complex formation. The latter is especially justified knowing that the structural comparison of the ACE2–spike protein binding region with MAO B resulted in approximately 90% structure overlap, despite only 51% structural similarity with the overall ACE2 structure [36]. Our results demonstrate that this recognition is particularly favourable for SA⋅⋅⋅MAO B, where the calculated binding energy surpasses that of SA⋅⋅⋅ACE2 by –7.9 kcal mol^−1^, thus offering an interesting insight and perspective.

### 3.3 Changes in the affinity of the MAO isoforms towards physiological substrates following an interaction with the SARS-CoV-2 variants

Lastly, we evaluated how the WT/SA⋅⋅⋅MAO complexes impact MAO activity through the affinity for their brain amines. In doing so, we considered phenylethylamine (**PEA**) for both MAO isoforms, in order to place our results in the context of experimental findings by Cuperlovic-Culf, Green and co-workers [36], and more specific amine neurotransmitters serotonin (**SER**) and dopamine (**DOP**), which are typical substrates for MAO A and MAO B, respectively. The calculated affinities are given in Table 2 and compared to relevant Michaelis-Menten constants, *K*_M_. We note in passing that, in the native MAO B, both subunits revealed comparable substrate affinities without any significant preference, in line with other reports [59], so a more exergonic binding is considered, while for the MAO B⋅⋅⋅WT/SA complexes, the results for both subunits are given, while we mostly discuss those pertaining to the subunits directly interacting with the spike protein.

**Table 2:** Changes in the binding affinity (Δ*G*_BIND_) between the MAO isoforms and their physiological substrates following a complex formation with the WT and SA SARS-CoV-2 variants (in kcal mol^−1^).^a^, ^b^

Substrate	MAO A	MAO A⋅⋅⋅WT	MAO A⋅⋅⋅SA	MAO B	MAO B⋅⋅⋅WT	MAO B⋅⋅⋅SA
	–16.8 ± 2.0 (*K*_M_ = 140 μM)	–17.0 ± 1.7	–15.8 ± 2.0	–12.0 ± 1.2 (*K*_M_ = 4 μM)	–9.4 ± 2.0 [–10.8 ± 2.1]	–14.8 ± 2.2 [–11.8 ± 1.5]
	–20.1 ± 1.8 (*K*_M_ = 137 μM)	–15.5 ± 2.1	–23.0 ± 1.6	–	–	–
	–	–	–	–20.7 ± 2.2 (*K*_M_ = 229 μM)	–15.4 ± 1.8 [–19.7 ± 1.2]	–23.0 ± 1.1 [–15.6 ± 1.0]

a Experimental *K*_M_ values are taken from ref. [60] and are given in round brackets.

b Results for the MAO B⋅⋅⋅WT/SA complexes pertain to the MAO B subunit directly interacting with the matching spike protein, while those for the other subunit are given in square brackets.

The results for native MAOs show excellent agreement with the *K*_M_ data (Table 2), which lends firm credence to the employed computational setup. Specifically, **PEA** prefers binding to MAO A over MAO B, in line with the experimental affinities [60]. Additionally, the latter translate to a difference of 2.1 kcal mol^−1^, which is well-matched by our computed affinity difference of 4.8 kcal mol^−1^ in favour of MAO A. Also, **DOP** is a better substrate for MAO B than **SER** is for MAO A, again tying in with experiments. There, an even stronger agreement between sets is achieved, as the experimental affinity difference of 0.3 kcal mol^−1^ between **DOP** and **SER** is almost perfectly reproduced by the computed value of 0.6 kcal mol^−1^.

When **PEA** is concerned, the effect of the WT on its MAO A affinity is modest, being only 0.2 kcal mol^−1^ higher. Yet, for MAO B, the impact is much more pronounced, which is particularly relevant, and the observed affinity reduction following the MAO B⋅⋅⋅WT complex formation is 2.6 kcal mol^−1^. The latter indicates about two orders of magnitude lower **PEA** binding, which will inevitably lead to a lower metabolic **PEA** conversion and higher **PEA** concentrations in infected individuals. This insight strongly agrees with the mentioned experiments [36], and helps explaining a detection of lower concentrations of **PEA** metabolites following a COVID-19 infection, thus mimicking the effects of the irreversible MAO inhibitor selegiline, whose application also increases brain **PEA** levels [61] that leads to oxidative stress [62], [63]. Still, we must emphasize that our results disagree with the suggestion that the spike protein is interfering with the substrate entrance into the MAO B active site [36]. The discussed binding poses in ref. [36] were obtained through docking simulations that did not explicitly consider neither the mitochondrial membrane nor the MAO B membrane bound regions [36], which artificially allowed the WT spike protein to reside in the area close to the membrane-mediated substrate entrances [59] that is otherwise inaccessible and occupied by the membrane. In contrast, our simulations included entire MAO structures immersed in an explicit membrane, and after a careful inspection of the obtained binding poses in the WT⋅⋅⋅MAO A/B complexes, we found no evidence of the spike protein blocking the known substrate entrances [59]. Instead, based on our current results, we propose that the spike protein is interfering with the MAO activity by modifying the electrostatic environment in the complex, a feature that we will come back to later in the text.

**Fig. 2:**
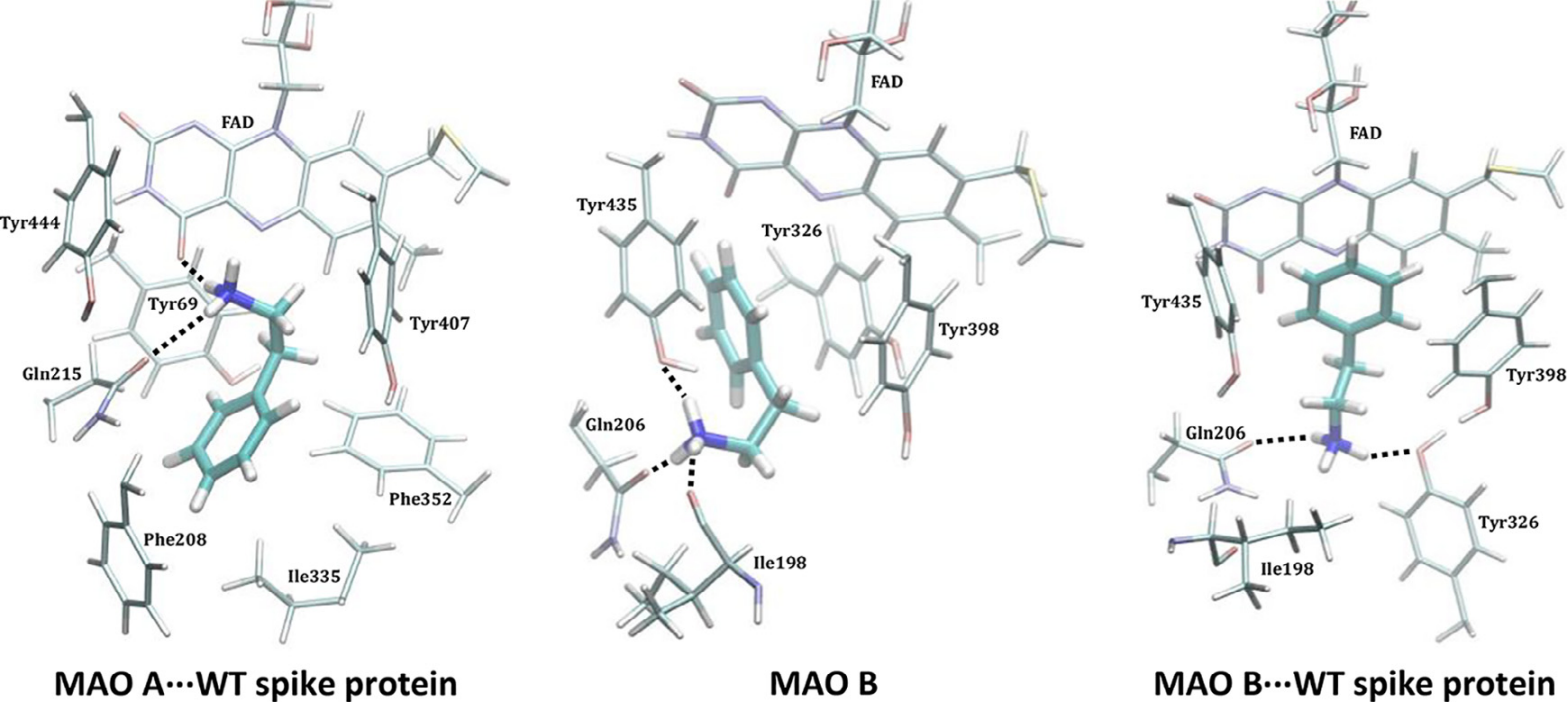
Binding position of **PEA** within the active sites of the MAO A⋅⋅⋅WT spike protein complex (left, similar in the native MAO A), native MAO B (middle) and the MAO B⋅⋅⋅WT spike protein complex (right) as obtained from MD simulations. The results for the MAO B⋅⋅⋅WT complexes pertain to the MAO B subunit directly interacting with the matching spike protein.

A practically unchanged **PEA** affinity for MAO A and the MAO A⋅⋅⋅WT complex comes as a result of a very similar **PEA** binding position in both instances (Fig. 2). There, its cationic amine forms a hydrogen bond with the Gln215 side chain and the carbonyl group of the FAD cofactor (Fig. S7), while its ethylphenyl unit engages in a series of aromatic C–H⋅⋅⋅π and π⋅⋅⋅π stacking interactions with Tyr407, Phe352, Tyr69 and Phe208 (Table S4). Such a binding pose is very much modified in MAO B, which is not surprising knowing that it is the predominant **PEA** metabolizing enzyme in the brain despite a lower affinity relative to MAO A [60]. There, **PEA** orients its aromatic unit towards FAD, and places its cationic amine within three hydrogen bonds: with the Tyr435 –OH group, the Gln206 side chain, and the Ile198 backbone (Fig. S8). This is further modulated in the MAO B⋅⋅⋅WT complex, which strongly favours hydrophobic π⋅⋅⋅π stacking interactions with FAD and C–H⋅⋅⋅π interactions with Tyr398 and Tyr435 (Fig. S9), at the expense of the hydrogen bonding contacts with –NH_3_^+^, which ultimately reduces the affinity.

Encouraged by rationalizing lower **PEA** metabolite concentrations upon the WT infection [36], we continued by analyzing a more specific MAO A and MAO B substrates, **SER** and **DOP**, respectively. **SER** is a typical MAO A substrate, and its affinity, Δ*G*_BIND_ = –20.1 kcal mol^−1^, comes as a result of strong hydrogen bonding between its protonated amine and both the FAD carbonyl group and the Gln215 side chain (Fig. S10), further supported by (i) the –OH hydrogen bonding with the Gly443 backbone and (ii) hydrophobic aromatic interactions with Tyr407 and Tyr444. This is significantly disrupted in MAO A⋅⋅⋅WT, and results in a different binding orientation (Fig. S11), where the mentioned three interaction motifs are replaced by the hydroxy –OH group and the protonated amine from **SER** interacting with the backbone amides of Asn181 and Ile207 (Fig. S12), respectively; the former becoming the most dominant pairing in that case (Table S4). This significantly lowers the **SER** affinity for MAO A⋅⋅⋅WT, being reduced by 4.6 kcal mol^−1^ to Δ*G*_BIND_ = –15.5 kcal mol^−1^. Such a large impact inevitably leads to a lower **SER** metabolism upon the WT infection, which strongly corroborates experimental measurements by Shen et al. [37]. On the other hand, **DOP** has the highest affinity among the studied amines, Δ*G*_BIND_ = –20.7 kcal mol^−1^, in line with its highest *K*_M_ value of 229 μM. Fascinatingly, in this case, the effect of the WT strain is also the greatest, evident in a 5.3 kcal mol^−1^ reduced affinity for MAO B. The latter is supported by a notable change in **DOP** orientation (Fig. S11), during which a range of hydrogen bonding contacts in the native MAO B (Fig. S13) are replaced by mostly aromatic C–H⋅⋅⋅π and π⋅⋅⋅π stacking interactions in the complex (Fig. S14). Therefore, as a conclusion, somewhat higher disturbances in the dopaminergic over serotonergic pathway could be expected following the WT variant infection, which agrees with the literature [13], [33].

**Fig. 3:**
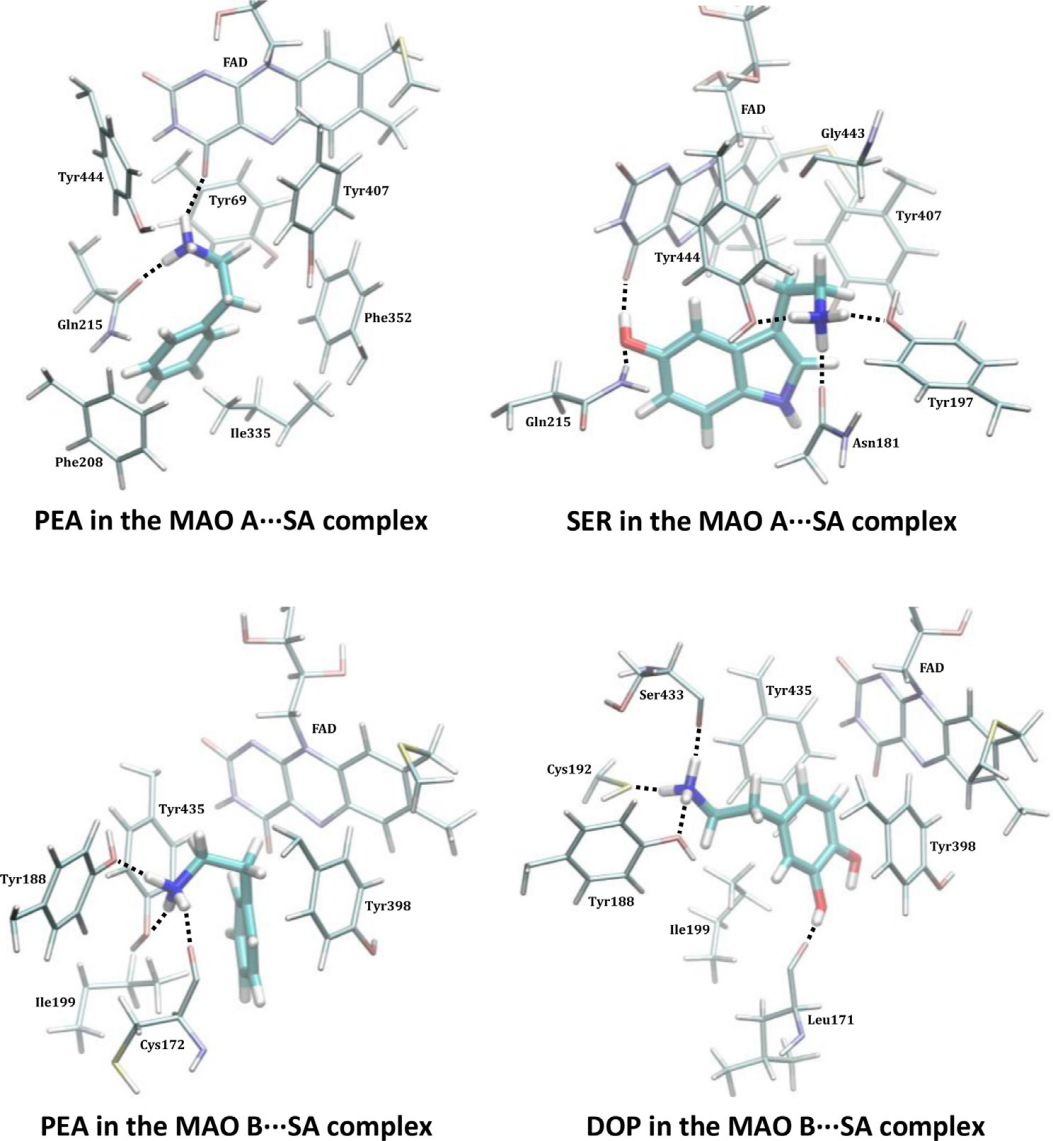
Binding positions of substrates within the active site of both MAO isoforms following the complex formation with the SARS-CoV-2 SA variant as obtained from the MD simulations. Identification of crucial interactions is presented in Figures S17–S20. The results for the MAO B⋅⋅⋅SA complexes pertain to the MAO B subunit directly interacting with the matching spike protein.

When a more contagious SA variant is concerned, it appears that its impact on both MAO isoforms is higher and more severe than that of the WT analogue (Fig. 3, Tables S4–S5), which parallels its effect on the ACE2 receptor. Therefore, in addition to causing more disturbances in the respiratory chain, an infection with the SA strain is likely to result in more problematic outcomes for the immediate and, especially, the long-term neurological conditions. Relative to the WT, the SA strain causes the affinity of the MAO substrates to significantly increase in all cases, except for **PEA** and MAO A, where it is only slightly reduced, by 1.0 kcal mol^−1^ to –15.8 kcal mol^−1^ (Table 2). This again suggests that **PEA** and MAO A are behaving differently relative to all other instances, and that the **PEA** pathway in the affected individuals will predominantly concern the MAO B enzyme, as it was also confirmed in the WT infection [36]. There, the affinity increases by 2.8 kcal mol^−1^ to –14.8 kcal mol^−1^ (Table S5), predominantly because of a favourable hydrogen bonding between its protonated amine and (i) the side chain hydroxy groups in Tyr435 and Tyr188, and (ii) the backbone amide in Cys172 (Fig. S19), where the interaction with the mentioned three residues carries 56% of the total affinity. With **SER**, the effect of the SA infection is the largest and its affinity for MAO A increases by 2.9 kcal mol^−1^ to –23.0 kcal mol^−1^. The latter follows a noteworthy change in the binding position in the SA⋅⋅⋅MAO A complex (Fig. 3), which allows **SER** a range of favourable and persistent hydrogen bonding contacts, including those with Gln215, Tyr444, Tyr197, Asn181 and the FAD cofactor (Fig. S18). These five MAO A residues, on their own, already contribute 17.2 kcal mol^−1^ to the binding energy, 75% in total, which is really striking. Such an affinity increase is analogously present in **DOP**, whose affinity for MAO B becomes 2.3 kcal mol^−1^ higher and equals that for **SER** and MAO A at –23.0 kcal mol^−1^. This is again preceded by a different **DOP** binding orientation that allows it to optimize hydrogen bonding contacts with Tyr188, Ser433, Leu171 and Cys192 (Fig. S20) that were all relatively insignificant for its binding in MAO B and MAO B⋅⋅⋅WT complex, which alone are responsible for a half of the total binding energy.

**Fig. 4:**
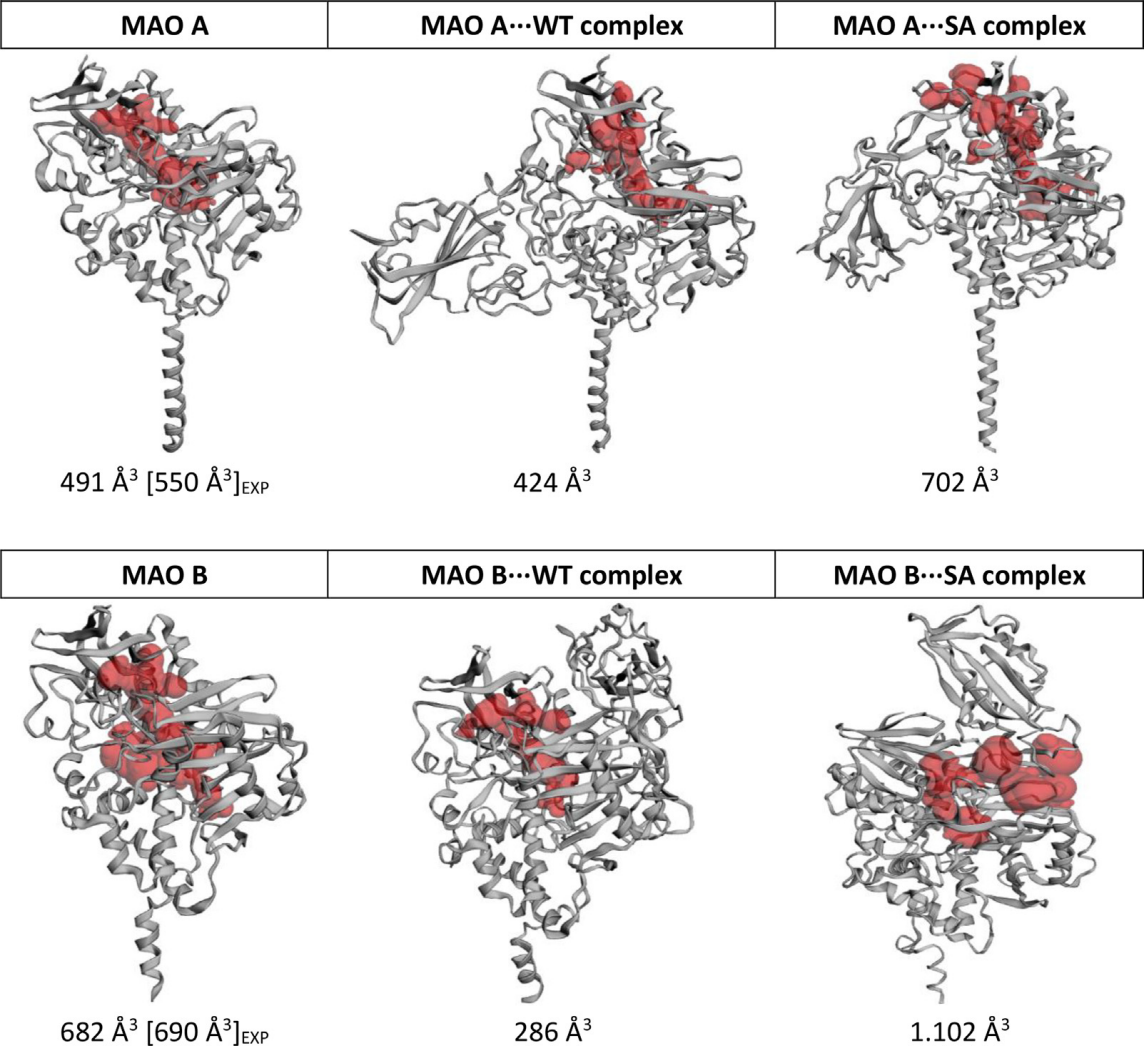
Solvent-accessible volume of MAO active sites (in red) in native forms and following the WT/SA complex formation, calculated with the CASTp online server and employing the radius probe of 1.5 Å. Experimental values for native enzymes are taken from ref. [65] and given in square brackets. For MAO B, only the subunit directly interacting with the spike protein is considered.

In order to confirm that the spike protein is interfering with MAO activity through changed electrostatic environment and to provide some evidence of a potential allosteric regulation, we have computed the solvent-accessible volume of the MAO active site using the CASTp online server [64], both in native forms and following the WT/SA complex formation (Fig. 4). The obtained results are found to be in excellent agreement with experiments for native enzymes through confirming a larger substrate cavity in MAO B [65] and tying in with the asymmetry in both MAO B subunits demonstrated earlier [59]. More importantly, the calculated values consistently predict a reduction in the active site volume in WT complexes, while, in contrast, reveal a volume enlargement in SA complexes. As demonstrated, these opposing trends lead to affinity decrease for typical substrates (**SER** and **DOP**) in both MAO⋅⋅⋅WT cases, while increasing it in the analogous MAO⋅⋅⋅SA complexes, which appears reasonable. Specifically, considering active site tyrosines belonging to the 'aromatic cage' perpendicular to the FAD cofactor as illustrative cases, Tyr407 and Tyr444 in MAO A, and Tyr398 and Tyr435 in MAO B (Fig. S21), a reduction in the active site volume in MAO⋅⋅⋅WT increases the average distance between hydroxyl oxygens in MAO A (from 6.4 to 6.7 Å), while drastically decreasing it in MAO B (from 6.9 and 7.3 to 4.5 Å for the subunit directly interacting with the spike protein). Given the demonstrated importance of these residues for substrate binding, both aspects, expectedly, decrease amine affinity. Contrary to that, in MAO⋅⋅⋅SA, an increase of the active site volume leaves inspected tyrosines intact, which allows for a better substrate placement and a more exergonic binding. Still, the described allosteric MAO regulation is likely small and less important relative to the electrostatic effect of the spike protein. A similar conclusion was reached by Darrell D. Mousseau et al. [66], who incubated both MAOs with Ca^2+^ and observed a selective increase in the MAO A activity, which the authors ascribed to an allosteric mechanism, yet the extent of the effect was modest and only approximately 20%, as measured by the increased H_2_O_2_ production in that case. While the effect on MAO B was negligible, the mentioned 20% increase translates to a cumulative effect of only 0.1 kcal mol^−1^, which gives some idea about the magnitude of the allosteric effect and suggests a modulated electrostatic environment as a likely dominant pathway for modified affinities.

The presented results raise a serious warning that, unlike a reduction in the metabolic conversion of neurotransmitters observed in the WT, the infection with the SA mutant strain will stimulate the metabolism of the investigated brain amines, which will result in their shortage. At the same time, this will increase the production of hydrogen peroxide and thereof derived reactive oxygen species (ROS), together with toxic aldehydes and ammonia, which are all by-products of the MAO-catalyzed amine degradation [67], [68]. Unfortunately, all of the mentioned metabolites, along with the subsequent inflammation pathways, can induce neurodegenerative processes on their own and can further assist in their progression.

## 4 Conclusions

A combination of docking and molecular dynamics simulations reveals that the spike protein from two SARS-CoV-2 variants, namely the wild type (WT) and the mutated B.1.351 South African (SA) strain, possess affinity towards monoamine oxidase enzymes (MAOs) that is comparable to that for its ACE2 receptor. This allows a formation of the corresponding WT/SA⋅⋅⋅MAO complexes following an initial respiratory infection, with the protein–protein recognition being analogously established predominantly via residues from the receptor-binding motif in all cases. Knowing that alterations in MAO activities are a potential foundation of oxidative stress and various neuropsychiatric disorders [29], [31], [67], such as Parkinson's or Alzheimer's disease [69], [70], the demonstrated feasibility of the WT/SA⋅⋅⋅MAO complex formation opens a possibility that the interference with the brain MAO activity is responsible for an increased development and faster progression of neurodegenerative illnesses in COVID-19 infected individuals [71]; a disturbing medical issue that is presently widely discussed in the literature.

Our computational results show that spike protein⋅⋅⋅MAO complexes significantly lower MAO affinities towards their neurotransmitter substrates in the WT infection, thus resulting in a reduced metabolic conversion, being firmly in line with the experimentally measured trends for **PEA** [36] and a range of other metabolites in mildly affected patients [37]. However, a more severe SA variant offers even more stable complexes with both MAO isoforms, which in the case of MAO B even surpasses the stability of the matching SA⋅⋅⋅ACE2 complex. Interestingly, this leads to an increase in the MAO affinity for its substrates and, consequently, higher rates of their metabolic degradation, a trend that firmly agrees with experiments on serotonin and thereof derived conclusion that “serotonin levels would further decrease as the severity of COVID-19 increases” [37]. The latter likely promotes neurological disturbances through the immediate overproduction of hydrogen peroxide, ROS and toxic aldehydes. In this context and within the possibility for new and more contagious mutant strains likely emerging in the near future [72], we firmly advise that the presented prospect for the SARS-CoV-2 induced neurological complications should be carefully monitored.

It is beyond doubt that, besides changing their enzymatic function, binding of the spike protein to the MAO enzymes can additionally alter several of their roles, such as post-translational modifications or associations with protein partners [73]. This is why a possibility that the SARS-CoV-2 influences MAO activity, thereby inducing neurological complications, requires further clinical investigations, which are currently scarce since most of the ongoing research focuses on drug design. Yet, our results are, to the best of our knowledge, the first in identifying a critical role of the MAO metabolic activity in this respect, therefore placing a neurobiological link between these two conditions in the spotlight, and issuing a warning that it should not be ignored. In addition, we hope our work will stimulate researchers to identify other biological systems that could be potential targets for the spike protein [74], which could also generate various disturbances in the infected patients. Some efforts in this direction have already been made [75].

Lastly, additional research is required to establish what effect clinically employed MAO inhibitors [76], [77] might have on these pathways as, currently, there is no evidence to support either the withholding or increasing MAO inhibitors in COVID-19 treatment.

## CRediT authorship contribution statement

**Lucija Hok:** Investigation, Data curation, Formal analysis, Visualization, Writing – review & editing. **Hrvoje Rimac:** Investigation, Data curation, Formal analysis, Writing – review & editing. **Janez Mavri:** Conceptualization, Formal analysis, Writing – review & editing, Project administration. **Robert Vianello:** Conceptualization, Formal analysis, Writing – original draft, Writing – review & editing, Supervision, Project administration, Funding acquisition.

## Declaration of Competing Interest

The authors declare that they have no known competing financial interests or personal relationships that could have appeared to influence the work reported in this paper.
